# Immunometabolic Mechanisms of Coronary Microvascular Dysfunction in Coronary Artery Disease: The Role of Mitochondrial Stress, Endothelial Senescence, and Regulated Cell Death

**DOI:** 10.3390/cells15131132

**Published:** 2026-06-23

**Authors:** Mateusz Lucki, Ewa Lucka, Przemysław Mitkowski, Maciej Lesiak

**Affiliations:** 1 Department of Cardiology, Poznan University of Medical Sciences, 61-701 Poznań, Poland; mateusz.lucki@ump.edu.pl (M.L.); przemyslaw.mitkowski@ump.edu.pl (P.M.); maciej.lesiak@ump.edu.pl (M.L.); 2 Clinical Rehabilitation Laboratory, Department of Rehabilitation and Physiotherapy, University of Medical Sciences, 60-545 Poznań, Poland

**Keywords:** coronary artery disease, chronic coronary syndromes, coronary microvascular dysfunction, endothelium, ferroptosis, immunometabolism, inflammation, microvascular angina, oxidative stress, pyroptosis

## Abstract

**Highlights:**

**What are the main findings?**

- Coronary microvascular dysfunction emerges as an immunometabolic vascular network disorder in coronary artery disease, where mitochondrial stress, NAD^+^ depletion, and redox imbalance converge to drive endothelial senescence and loss of vasodilatory capacity.
- Emerging evidence suggests that pyroptosis and ferroptosis may act as important amplifiers of microvascular injury by linking inflammasome activation, iron-dependent lipid peroxidation, and impaired cellular repair mechanisms, thereby contributing to persistent endothelial dysfunction and inflammation.

**What are the implications of the main findings?**

- These findings identify immunometabolic reprogramming and regulated cell death as promising therapeutic targets, supporting the development of future disease-modifying strategies for patients with microvascular angina and chronic coronary syndromes.
- Coronary flow reserve (CFR/MFR) represents a clinically translatable systems-level biomarker that integrates perturbations with functional microvascular impairment and may improve patient phenotyping, risk stratification, and therapeutic guidance.

**Abstract:**

Chronic coronary syndromes (CCSs) are increasingly recognized as complex immunometabolic vascular disorders in which coronary microvascular dysfunction (CMD), persistent low-grade inflammation, oxidative stress, and maladaptive cellular remodeling contribute to ischemic symptoms and adverse outcomes beyond epicardial stenosis. CMD represents a heterogeneous condition comprising both functional and structural endotypes and constitutes a major determinant of myocardial ischemia, heart failure progression, and adverse cardiovascular outcomes, even in the absence of obstructive coronary artery disease. Emerging evidence indicates that immunometabolic reprogramming of endothelial cells, vascular smooth muscle cells, and immune cells sustains microvascular dysfunction in CCSs. Metabolic shifts toward glycolysis, mitochondrial dysfunction, redox imbalance, and dysregulated lipid metabolism promote chronic inflammatory activation within the coronary microenvironment. Convergent mitochondrial stress (including NAD^+^ decline) and redox injury promote endothelial senescence and increase susceptibility to regulated cell death, progressively limiting vasodilatory reserve and predisposing to microvascular rarefaction. Pyroptosis and ferroptosis-like lipid peroxidation further exacerbate endothelial barrier disruption and inflammatory amplification. In parallel, inflammasome activation, iron-dependent lipid peroxidation, impaired autophagy, and endoplasmic reticulum stress form interconnected molecular networks that amplify vascular injury through self-reinforcing mechanisms. This narrative review integrates mechanistic and translational evidence linking immunometabolic dysregulation, mitochondrial stress, thromboinflammatory signaling, endothelial senescence, and regulated cell death to distinct CMD endotypes. We propose a systems-level framework in which coronary microvascular dysfunction is conceptualized as an immunometabolic vascular network disorder, with reduced coronary flow reserve (CFR)—often termed myocardial flow reserve (MFR) in PET studies—emerging as the integrative functional endpoint of these interacting molecular perturbations and a robust predictor of major cardiovascular events.

## 1. Introduction

Chronic coronary syndromes (CCSs) are increasingly recognized as complex systemic disorders that extend beyond simple epicardial atherosclerosis [1]. While obstructive coronary artery disease remains a major cause of myocardial ischemia, a substantial proportion of patients present with anginal symptoms or objective evidence of ischemia despite non-obstructive coronary arteries. This clinical scenario highlights the importance of coronary microvascular dysfunction (CMD) as a key contributor to myocardial ischemia and adverse cardiovascular outcomes [2,3,4].

CMD encompasses structural and functional alterations in the coronary microcirculation, including endothelial dysfunction, impaired vasodilatory capacity, abnormal vasomotor tone, capillary rarefaction, perivascular fibrosis, and microvascular obstruction [2]. These abnormalities may occur independently of epicardial stenosis and can significantly impair myocardial perfusion and oxygen delivery [5]. Increasing recognition of ischemia with non-obstructive coronary arteries (INOCA) and related conditions has therefore shifted attention toward the coronary microcirculation as a clinically relevant therapeutic target [3,4].

Coronary flow reserve (CFR)—often termed myocardial flow reserve (MFR) in positron emission tomography (PET) studies—represents the ratio of hyperemic to resting myocardial blood flow and provides an integrative functional measure of coronary vasomotor capacity across both epicardial and microvascular compartments [6,7]. Reduced CFR has consistently been associated with adverse cardiovascular outcomes independent of epicardial stenosis severity, underscoring the prognostic relevance of coronary microvascular dysfunction.

Beyond abnormalities in vascular tone, growing evidence indicates that CMD is sustained by persistent inflammatory activation, metabolic dysregulation, and oxidative stress within the coronary microenvironment [2,8]. Endothelial cells, vascular smooth muscle cells, macrophages, perivascular fibroblasts, platelets, and circulating immune cells form an interactive cellular network in which chronic low-grade inflammation promotes endothelial activation, leukocyte recruitment, and microvascular injury [9].

Recent advances in cardiovascular immunology and metabolism have highlighted immunometabolic reprogramming as a key mechanism linking cellular metabolic pathways with inflammatory signaling in vascular disease [10]. In endothelial cells and activated macrophages, metabolic shifts toward aerobic glycolysis promote pro-inflammatory cytokine production through HIF-1α-dependent transcriptional programs [11]. Concurrent mitochondrial dysfunction and excessive reactive oxygen species (ROS) generation reduce nitric oxide (NO) bioavailability and activate redox-sensitive inflammatory pathways, further amplifying endothelial injury and microvascular dysfunction [12].

Nutrient-sensing pathways—including mTOR, AMPK, and sirtuin signaling—coordinate cellular responses to metabolic stress and regulate endothelial adaptation to environmental cues [13]. Dysregulation of these pathways contributes to oxidative stress, inflammatory signaling, and metabolic inflexibility, creating a permissive environment for persistent microvascular injury and regulated cell death pathways [14,15,16].

Systemic comorbidities such as diabetes mellitus, obesity, chronic kidney disease, and autoimmune disorders further intensify these mechanisms by amplifying oxidative stress, inflammatory signaling, and endothelial dysfunction [17,18]. These conditions therefore act as systemic accelerators of CMD progression and contribute to the heterogeneity of clinical presentations observed in CCSs.

Taken together, these observations support a conceptual shift in which CCSs are increasingly viewed as systemic vascular disorders involving complex interactions between metabolic stress, inflammation, mitochondrial dysfunction, and microvascular injury. In this narrative review, we synthesize mechanistic and translational evidence linking immunometabolic dysregulation, redox imbalance, mitochondrial stress, endothelial senescence, and regulated cell death pathways to coronary microvascular dysfunction. We further propose an integrative systems-level framework that connects these molecular perturbations with impaired coronary flow reserve and long-term clinical manifestations of chronic coronary syndromes.

## 2. Review Methodology

This narrative review was conducted in accordance with the SANRA framework to ensure methodological transparency and internal consistency [19]. The objective was to synthesize current mechanistic and translational evidence linking immunometabolic reprogramming, oxidative stress, mitochondrial dysfunction, regulated cell death, and CMD within CCSs.

To enhance methodological transparency, studies were qualitatively classified as high, moderate, or exploratory evidence according to translational relevance, methodological rigor, and direct applicability to coronary microvascular dysfunction. Of the 69 cited references, one publication referred to the SANRA methodology and was not included in evidence grading [19]. The remaining 83 references were categorized as high-level clinical/translational evidence (*n* = 21), moderate translational evidence (*n* = 29), or exploratory mechanistic evidence (*n* = 33). High-level evidence included clinical guidelines, consensus documents, systematic reviews and meta-analyses, prospective outcome studies, and human imaging studies assessing coronary flow reserve (CFR) or myocardial flow reserve (MFR). Moderate evidence included clinically oriented mechanistic reviews, disease-specific translational studies, and human pathophysiological investigations. Exploratory evidence included experimental, animal, cellular, molecular, and pathway-focused studies used primarily to support mechanistic plausibility. This classification was used to guide evidence weighting during narrative synthesis and interpretation.

A structured literature search was performed in PubMed/MEDLINE, Web of Science, and Scopus covering publications from January 2000 to June 2026. Seminal earlier studies were included when necessary to provide mechanistic context. Representative search strategies combined terms related to coronary microvascular dysfunction (“coronary microvascular dysfunction”, “microvascular angina”, “INOCA”, “coronary flow reserve”) with immunometabolic and cell-death pathways (“immunometabolism”, “metabolic reprogramming”, “HIF-1α”, “mTOR”, “AMPK”, “inflammasome”, “pyroptosis”, “ferroptosis”, “necroptosis”). Reference lists of selected articles were manually screened to identify additional relevant publications.

Eligible studies were required to provide mechanistic insight into coronary microvascular biology, examine immunometabolic, redox, mitochondrial, inflammatory, or regulated cell death pathways relevant to vascular pathology, or report translational and clinical evidence linking CMD—particularly coronary flow reserve (CFR/MFR)—to outcomes. Non-peer-reviewed abstracts, studies lacking mechanistic relevance, and publications focused exclusively on epicardial stenosis without microvascular consideration were excluded.

Given the heterogeneity of available data, evidence was interpreted within biological and methodological context. Human clinical data, particularly prospective outcome studies and imaging-based assessments of CFR, were prioritized over experimental findings. Experimental in vivo and in vitro studies were integrated to support mechanistic plausibility but were not extrapolated beyond their translational scope. Where direct human validation was limited—such as in the case of ferroptosis in coronary microvessels—conclusions were cautiously framed as mechanistic vulnerability rather than definitive pathway attribution.

This approach enabled a systems-level integration of molecular, cellular, and clinical evidence while maintaining appropriate weighting of translational strength and avoiding overinterpretation of experimental data.

## 3. Molecular Basis of Coronary Microvascular Dysfunction

Coronary microvascular dysfunction arises from the convergence of multiple molecular perturbations that extend beyond isolated abnormalities in endothelial nitric oxide signaling. Redox imbalance, mitochondrial bioenergetic failure, immunometabolic reprogramming, endoplasmic reticulum stress, regulated cell death pathways, and thromboinflammatory signaling do not operate as independent mechanisms but interact within a dynamic and self-reinforcing network at the level of the coronary microcirculation.

Endothelial cells function as central integrative hubs, translating systemic metabolic stress and immune activation into structural remodeling, capillary rarefaction, barrier dysfunction, and impaired vasomotor reserve. Importantly, these molecular programs interact within an interconnected regulatory network that ultimately converges on impaired CFR, which serves as an integrative functional readout of coronary microvascular performance.

**Figure 1:**
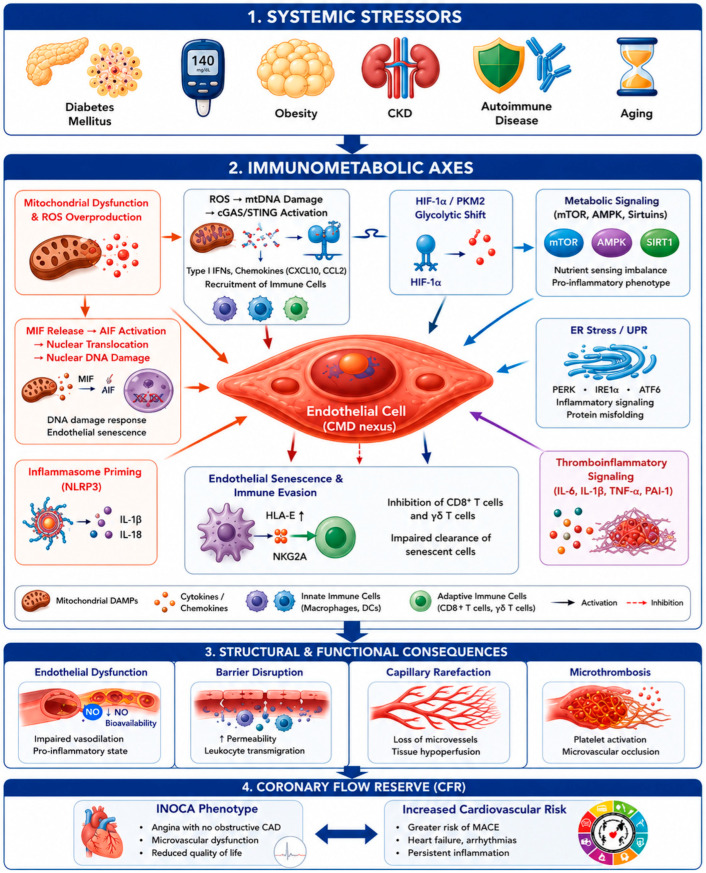
Integrated Immunometabolic Network Driving Coronary Microvascular Dysfunction. Systemic stressors including diabetes mellitus, obesity, chronic kidney disease, autoimmune diseases, and aging promote coronary microvascular dysfunction through interconnected immunometabolic pathways. Mitochondrial dysfunction, oxidative stress, cGAS–STING activation, metabolic reprogramming, endoplasmic reticulum stress, inflammasome activation, endothelial senescence, immune evasion, and thromboinflammatory signaling converge on the coronary microvascular endothelium, resulting in endothelial dysfunction, barrier disruption, capillary rarefaction, microthrombosis, impaired coronary flow reserve, and the clinical INOCA phenotype. AIF—apoptosis-inducing factor; AMPK—AMP-activated protein kinase; ATF6—activating transcription factor 6; CCL2—C-C motif chemokine ligand 2; cGAS—cyclic GMP-AMP synthase; CD8+—CD8-positive T lymphocytes; CFR—coronary flow reserve; CKD—chronic kidney disease; CMD—coronary microvascular dysfunction; CXCL10—C-X-C motif chemokine ligand 10; ER—endoplasmic reticulum; HIF-1α—hypoxia-inducible factor-1α; HLA-E—human leukocyte antigen E; IFNs—interferons; IL-1β—interleukin-1β; IL-6—interleukin-6; IL-18—interleukin-18; INOCA—ischemia with non-obstructive coronary arteries; IRE1α—inositol-requiring enzyme 1 alpha; MACE—major adverse cardiovascular events; MIF—macrophage migration inhibitory factor; mTOR—mechanistic target of rapamycin; mtDNA—mitochondrial DNA; NKG2A—natural killer group 2A receptor; NLRP3—NOD-like receptor family pyrin domain containing 3; NO—nitric oxide; PAI-1—plasminogen activator inhibitor-1; PERK—protein kinase RNA-like endoplasmic reticulum kinase; PKM2—pyruvate kinase M2; ROS—reactive oxygen species; SIRT1—sirtuin 1; STING—stimulator of interferon genes; TNF-α—tumor necrosis factor-α; UPR—unfolded protein response; γδ T cells—gamma delta T lymphocytes.

To provide a systems-level overview of these interconnected pathways before examining each axis in detail, Figure 1 summarizes the integrated immunometabolic and redox–cell death network that underlies coronary microvascular dysfunction in chronic coronary syndromes.

### 3.1. Endothelial Nitric Oxide Signaling, Redox Homeostasis, and Oxidative Stress

Endothelial cells play a central role in maintaining coronary microvascular tone, primarily through the production of nitric oxide (NO) via endothelial nitric oxide synthase (eNOS) [20]. NO mediates vasodilation, inhibits platelet aggregation, and suppresses leukocyte adhesion and smooth muscle proliferation, collectively preserving microvascular integrity [21].

Efficient eNOS activity depends on phosphorylation at Ser1177 through PI3K–Akt signaling and calcium–calmodulin regulation, enabling effective L-arginine oxidation in the presence of tetrahydrobiopterin (BH4) [20]. In CMD, endothelial dysfunction often arises from eNOS uncoupling, which is promoted by BH4 depletion, accumulation of asymmetric dimethylarginine (ADMA), and S-glutathionylation. Uncoupled eNOS generates superoxide instead of NO, further exacerbating oxidative stress [12].

Major sources of reactive oxygen species (ROS) include NADPH oxidases and dysfunctional mitochondria [12,22]. Inflammatory activation and disturbed shear stress contribute to oxidative stress, while impaired antioxidant defenses amplify redox imbalance [22,23,24,25]. The resulting oxidative stress reduces NO bioavailability, impairs endothelium-dependent vasodilation, and promotes inflammatory endothelial activation [12,24].

### 3.2. Mechanotransduction and Cytoskeletal Remodeling

Coronary microvessels are continuously exposed to biomechanical forces, such as laminar shear stress and cyclic stretch, which modulate endothelial phenotype and function [26]. Laminar shear stress activates mechanosensory signaling leading to PI3K–Akt activation, eNOS phosphorylation, and KLF2/KLF4-mediated anti-inflammatory transcription [27,28].

Disturbed or oscillatory flow reduces endothelial protective transcriptional programs, decreases NO bioavailability, and enhances inflammatory signaling [26]. Mechanosensitive ion channels regulate intracellular calcium influx and endothelial adaptation to shear; their dysregulation can impair eNOS activation and contribute to endothelial dysfunction [26]. Piezo1 is a key mechanosensor integrating physiological force and vascular responses, supporting the concept that altered mechanosensing may contribute to microvascular disease phenotypes [29].

Integrin–FAK signaling connects extracellular matrix cues to cytoskeletal remodeling and MAPK pathway activation, thereby linking disturbed mechanosensing with endothelial dysfunction and impaired coronary flow reserve [17,26].

Together, impaired NO signaling, oxidative stress, and disturbed mechanotransduction establish a permissive environment for the immunometabolic and regulated cell death pathways discussed in subsequent sections.

### 3.3. Coronary Microvascular Rarefaction and Structural Alterations

CMD is often accompanied by structural remodeling of the microcirculation, including capillary rarefaction, perivascular fibrosis, and thickening of the basement membrane [2]. Capillary loss reduces perfusion reserve, while perivascular fibrosis impairs vasodilation and increases vascular stiffness [4].

Inflammatory mediators, ROS, and metabolic stress collectively promote apoptosis, inflammasome-related injury, and ferroptosis-like vulnerability in endothelial and perivascular cells, contributing to rarefaction [14]. Dysregulated extracellular matrix turnover further disrupts vascular architecture and microvascular integrity [26].

In addition to endothelial cell loss, increasing evidence suggests that microvascular thrombosis contributes directly to capillary rarefaction. Endothelial dysfunction promotes a prothrombotic phenotype characterized by increased expression of tissue factor, PAI-1, adhesion molecules, and inflammatory cytokines such as IL-6 and TNF-α. These alterations facilitate platelet activation, leukocyte recruitment, and formation of microthrombi within the coronary microcirculation. Persistent or recurrent capillary obstruction may result in local ischemia, endothelial injury, and eventual disappearance of non-perfused microvessels, thereby reducing capillary density. Thus, microthrombosis is not merely a consequence of endothelial dysfunction but represents an active driver of capillary rarefaction and adverse microvascular remodeling [30,31,32,33].

These structural alterations are associated with impaired myocardial perfusion, heightened ischemic susceptibility, and adverse remodeling, even in the absence of epicardial stenoses [6].

Collectively, these molecular and structural alterations reduce perfusion reserve and create a permissive milieu for immune recruitment, metabolic stress responses, and maladaptive thromboinflammatory signaling. This provides a mechanistic bridge from microvascular biology to immunometabolic reprogramming discussed in the next section [2,6].

## 4. Immunometabolic Reprogramming

### 4.1. Glycolytic Switching and TCA Rewiring

In CMD, endothelial cells and macrophages undergo profound metabolic reprogramming, shifting from oxidative phosphorylation to aerobic glycolysis, a phenomenon similar to the “Warburg effect” observed in cancer cells [11]. Upregulation of key glycolytic enzymes supports ATP production under stress conditions while promoting pro-inflammatory signaling via HIF-1α and STAT3 pathways [10].

Disruption of the tricarboxylic acid (TCA) cycle leads to accumulation of succinate, which inhibits prolyl hydroxylases, stabilizing HIF-1α even under normoxic conditions [34]. Stabilized HIF-1α induces transcription of IL-1β and other inflammatory mediators, reinforcing glycolytic metabolism and creating a feed-forward inflammatory loop [34].

Succinate oxidation further amplifies inflammatory signaling and promotes endothelial and macrophage activation through HIF-1α-dependent mechanisms [34]. Additional links between glycolytic reprogramming, mitochondrial dysfunction, and oxidative stress are discussed in Section 4.4.

Enhanced glycolysis promotes endothelial activation and leukocyte recruitment, linking metabolic reprogramming with microvascular inflammation in CMD [10].

### 4.2. mTOR, AMPK, and Sirtuin Signaling

Cellular nutrient-sensing pathways, including mechanistic target of rapamycin (mTOR), AMP-activated protein kinase (AMPK), and sirtuins, orchestrate the response to metabolic stress in the coronary microcirculation [13]. Hyperactive mTORC1 promotes anabolic growth, sustains glycolytic flux, and suppresses adaptive catabolic programs, fostering a pro-inflammatory endothelial and macrophage phenotype [13].

Conversely, AMPK activation supports metabolic flexibility and endothelial resilience, promoting adaptive responses to energy stress [35]. Suppression of AMPK in metabolic disorders contributes to oxidative stress, endothelial dysfunction, and heightened susceptibility to cellular injury [17].

Sirtuin biology is closely tied to NAD^+^ metabolism and cellular stress resistance. NAD^+^ depletion in chronic metabolic disease can compromise mitochondrial function and stress adaptation, lowering the threshold for regulated cell death pathways [36,37].

Beyond their metabolic functions, sirtuins also participate in immune surveillance pathways. SIRT1 has been shown to regulate the expression of stress-induced ligands recognized by the activating receptor NKG2D on natural killer (NK) cells and cytotoxic T lymphocytes. Through modulation of these stress signals, sirtuins may influence the immune-mediated clearance of damaged or senescent endothelial cells. Impaired SIRT1 activity in metabolically stressed tissues may therefore contribute not only to endothelial dysfunction but also to defective immune surveillance and accumulation of senescent cells, reinforcing chronic inflammation and microvascular remodeling [38]. NAD^+^ metabolism therefore links energetic state, redox balance, and cellular resilience relevant to CMD [36].

### 4.3. Lipid Metabolism and Oxidative Lipotoxicity

Altered lipid handling in endothelial cells and macrophages contributes to CMD through inflammatory activation, endothelial dysfunction, and vascular remodeling [9]. Intracellular lipid accumulation can induce endoplasmic reticulum (ER) stress, activate the unfolded protein response (UPR), and promote oxidative stress, increasing susceptibility to cell injury and death [39].

Lipid peroxidation under conditions of oxidative stress and iron availability increases vulnerability to ferroptosis-like injury [15,16]. In addition, interactions between lipid sensing pathways and innate immune signaling amplify inflammatory responses and endothelial dysfunction [9,12].

### 4.4. Mitochondrial Dysfunction and Bioenergetic Failure

In CMD, mitochondrial dysfunction acts as a central bioenergetic and signaling hub. Impaired oxidative phosphorylation and defective mitochondrial quality control increase mitochondrial reactive oxygen species (ROS), reducing nitric oxide bioavailability and sustaining endothelial inflammatory activation [12,24]. Beyond ATP depletion, mitochondrial stress promotes endothelial senescence and increases susceptibility to regulated cell death, thereby contributing to functional impairment and, over time, structural microvascular rarefaction [36,37,40,41].

Dynamic mitochondrial remodeling further contributes to endothelial vulnerability. Excessive mitochondrial fission, mediated in part by dynamin-related protein 1 (DRP1), disrupts mitochondrial networking, impairs bioenergetic flexibility, and enhances ROS production [42,43]. Experimental data indicate that altered mitochondrial dynamics promote endothelial inflammatory activation, establishing a mechanistic bridge between metabolic stress and vascular dysfunction [43].

Bioenergetic failure, including reduced ATP availability and NAD^+^ depletion, compromises stress adaptation, antioxidant defense, and sirtuin-dependent mitochondrial maintenance pathways [36,37]. Suppressed energy-sensing and mitochondrial homeostasis mechanisms lower the threshold for endothelial senescence and regulated cell death, thereby promoting capillary rarefaction and persistent reduction in CFR [35,36].

Importantly, defective mitophagy may permit the accumulation of damaged mitochondria and the release of mitochondrial DNA (mtDNA) into the cytosol, where it can engage innate DNA-sensing pathways such as the cyclic GMP–AMP synthase–stimulator of interferon genes (cGAS–STING) axis. Experimental evidence suggests that activation of cGAS–STING signaling may provide a mechanistic link between mitochondrial quality-control failure and sustained vascular inflammatory signaling, with downstream consequences for endothelial dysfunction and senescence-associated programs [44,45]. Through this mechanism, mitochondrial stress extends beyond metabolic insufficiency to become a driver of persistent immunometabolic activation in CMD.

Excessive mitochondrial ROS generation plays a central role in this process because mitochondrial DNA (mtDNA) is located in close proximity to the electron transport chain and lacks the protective histone structures characteristic of nuclear DNA. Consequently, oxidative stress readily induces mtDNA damage and fragmentation. Released mtDNA acts as a damage-associated molecular pattern (DAMP), activating innate immune pathways, particularly the cGAS–STING signaling axis. Activation of cGAS–STING promotes type I interferon responses, NF-κB signaling, and chemokine production, thereby facilitating recruitment of inflammatory cells and sustaining vascular inflammation. Through these mechanisms, mitochondrial dysfunction extends beyond bioenergetic impairment and becomes a direct driver of chronic immunometabolic activation [46,47].

Mitochondrial injury may additionally promote endothelial senescence through the release of mitochondrial proteins into the cytoplasm. Experimental evidence suggests that macrophage migration inhibitory factor (MIF) released from damaged mitochondria can interact with apoptosis-inducing factor (AIF), facilitating nuclear translocation and DNA damage responses that reinforce cellular senescence programs. These observations further support the concept that mitochondrial dysfunction links metabolic stress with immune activation and vascular aging [47,48].

Collectively, mitochondrial dysfunction in CMD represents not merely an energetic deficit but a signaling hub that integrates redox imbalance, innate immune activation, endothelial senescence, and regulated cell death, thereby contributing to both functional microvascular impairment and structural rarefaction.

### 4.5. Endothelial Senescence as a Convergence Node of Immunometabolic Stress

Persistent mitochondrial dysfunction, NAD^+^ depletion, oxidative stress, and chronic inflammatory priming promote the emergence of endothelial senescence as a critical convergence mechanism in coronary microvascular dysfunction [3,12,24,37,40]. Senescent endothelial cells exhibit stable cell-cycle arrest mediated by activation of the p53–p21 and p16^INK4a–Rb pathways, accompanied by impaired proliferative and reparative capacity [1,40].

At the molecular level, mitochondrial ROS overproduction and DNA damage signaling activate p53-dependent transcriptional programs, while sustained metabolic stress and epigenetic alterations reinforce p16^INK4a expression [24,40]. NAD^+^ decline further compromises sirtuin-dependent genomic stability and mitochondrial quality control, lowering the threshold for senescence commitment [36,37,41].

Senescent endothelial cells acquire a senescence-associated secretory phenotype (SASP), characterized by secretion of IL-6, IL-1β, TNF-α, matrix metalloproteinases, and pro-thrombotic mediators, thereby reinforcing inflammatory signaling and redox imbalance [9,40,41]. This SASP milieu amplifies local inflammation, oxidative stress, extracellular matrix remodeling, and microvascular rarefaction [2,39,40]. We propose that endothelial senescence may represent a transitional checkpoint between reversible functional microvascular dysregulation and irreversible structural rarefaction, marking the tipping point from adaptive metabolic stress responses to persistent microvascular failure.

Available evidence suggests that mechanisms responsible for immune-mediated clearance may become progressively impaired [49,50]. Emerging evidence indicates that senescent endothelial cells may upregulate HLA-E expression, which interacts with inhibitory NKG2A receptors expressed on cytotoxic CD8^+^ T lymphocytes and γδ T cells [49,51]. This immune-evasion mechanism attenuates cytotoxic responses and promotes accumulation of senescent cells within the vascular wall despite ongoing immune surveillance [49,50,52].

Beyond serving as markers of vascular aging, senescent endothelial cells actively contribute to disease progression through the senescence-associated secretory phenotype (SASP). In addition to IL-1β and TNF-α, IL-6 appears particularly important because it promotes thromboinflammatory signaling, endothelial activation, leukocyte recruitment, and vascular remodeling. Consequently, endothelial senescence establishes a self-perpetuating cycle in which impaired immune clearance and persistent SASP production maintain chronic inflammation, oxidative stress, and progressive microvascular dysfunction [40,41,53,54].

Importantly, endothelial senescence links immunometabolic reprogramming with structural microvascular deterioration: glycolytic switching and mitochondrial dysfunction do not merely impair vasomotor tone but progressively limit regenerative capacity, thereby promoting capillary loss and persistent reduction in coronary flow reserve [2,35,36,40].

Thus, endothelial senescence may represent a mechanistic bridge between reversible functional CMD and structurally predominant endotypes characterized by microvascular rarefaction and impaired hyperemic reserve [2,6,40].

Beyond local endothelial injury, senescence-associated inflammatory signaling may propagate vascular dysfunction across compartments through thromboinflammatory activation and extracellular vesicle-mediated intercellular communication, linking metabolic stress with systemic vascular inflammation.

## 5. Regulated Cell Death Pathways in Coronary Microvascular Dysfunction

### 5.1. Pyroptosis: Inflammasome-Driven Injury

Pyroptosis is a lytic, pro-inflammatory form of programmed cell death mediated by inflammasomes [14]. Activation typically requires priming through inflammatory signaling and a secondary stress signal such as mitochondrial ROS, ionic flux, lysosomal dysfunction, or release of mitochondrial danger signals [14].

Assembly of inflammasome platforms leads to caspase activation, cleavage of pro-IL-1β and pro-IL-18, and processing of gasdermin family proteins. Gasdermin D (GSDMD) is considered the canonical executor of pyroptosis, and its N-terminal fragment forms membrane pores, causing cell swelling, membrane rupture, and release of pro-inflammatory cytokines [14,55]. Emerging evidence suggests that additional gasdermin family members, including GSDME, may also contribute to inflammatory endothelial injury [56].

In endothelial cells, pyroptotic signaling disrupts barrier integrity, promotes leukocyte recruitment, and contributes to microvascular inflammation [14]. Metabolic and mitochondrial pathways discussed in previous sections further potentiate inflammasome activation and inflammatory amplification [24,34]. Recurrent pyroptotic signaling may therefore contribute to the persistence of vascular inflammation and endothelial dysfunction in CMD [2].

While most mechanistic data is derived from experimental vascular models, indirect clinical evidence supporting inflammatory pathway modulation in cardiovascular disease suggests that inflammasome-linked signaling represents a biologically plausible contributor to CMD phenotypes [57].

### 5.2. Ferroptosis: Iron-Dependent Lipid Peroxidation

Ferroptosis is an iron-dependent form of regulated cell death characterized by accumulation of lipid peroxides [15]. Susceptibility is governed by antioxidant systems that constrain lipid peroxidation; impairment of these defenses permits uncontrolled peroxidation and membrane destabilization [16].

Inflammation and altered iron handling intersect to increase intracellular redox-active iron, amplifying oxidative lipid injury and potentially lowering the threshold for ferroptosis [58]. Ferroptotic injury is characterized by oxidative damage centered on lipid membranes and redox collapse, making it highly relevant to settings of chronic oxidative stress [16]. Lipid peroxidation products can also reinforce inflammatory signaling, creating bidirectional amplification between ferroptosis-like injury and vascular inflammation [12].

Emerging evidence indicates that cytotoxic immune cells may actively participate in the induction of ferroptotic cell death. Activated CD8+ T lymphocytes and NK cells release cytokines, including interferon-γ (IFN-γ), which can suppress cellular antioxidant defenses, impair cystine uptake, and promote lipid peroxidation. Through these mechanisms, immune-mediated cytotoxicity may lower the threshold for ferroptosis and amplify tissue injury under conditions of chronic inflammation. This highlights an important intersection between immune surveillance, inflammatory signaling, and regulated cell death pathways relevant to coronary microvascular dysfunction [59,60,61].

In CMD, direct histological confirmation of ferroptotic cell death within human coronary microvessels is lacking. Current human data largely reflect lipid peroxidation markers and altered iron-handling signatures rather than definitive demonstration of GPX4 failure or ferroptotic morphology. Thus, the most defensible interpretation is the presence of ferroptosis-like vulnerability under chronic oxidative stress conditions [15,16].

Crosstalk between ferroptosis and inflammasome-associated pathways can amplify vascular injury: oxidative lipid stress primes inflammatory responses, while inflammatory cytokines enhance oxidative stress and alter iron metabolism [14]. However, most evidence supporting this interaction originates from experimental studies, and its clinical significance in CMD requires further validation [14,15,16].

### 5.3. Necroptosis and Apoptosis

Necroptosis is a programmed, caspase-independent form of necrosis mediated by RIPK1–RIPK3–MLKL signaling [62]. Necroptotic cell death releases damage-associated molecular patterns (DAMPs), amplifying sterile inflammation and potentially sustaining microvascular inflammatory activation. However, direct evidence of necroptosis in human coronary microvascular tissue in CMD remains limited, and its contribution should currently be viewed as mechanistically plausible rather than definitively established.

Apoptosis contributes to chronic endothelial attrition and microvascular rarefaction. Oxidative and ER stress activate apoptotic signaling programs and compromise reparative capacity over time [39]. Although less overtly inflammatory than pyroptosis or necroptosis, cumulative apoptosis reduces microvascular integrity and contributes to structural deterioration of the coronary microcirculation [2].

### 5.4. ER Stress and Unfolded Protein Response Integration

ER stress is a key mediator connecting metabolic overload to cell death pathways. Activation of PERK, IRE1α, and ATF6 initially restores proteostasis through the unfolded protein response (UPR), but chronic ER stress induces apoptosis and inflammatory signaling programs [39].

ER stress disrupts redox homeostasis, promotes oxidative stress, and sensitizes cells to multiple regulated cell death pathways in chronic disease contexts [39]. Persistent UPR activation lowers the threshold for cumulative endothelial loss, capillary rarefaction, and impaired coronary flow reserve, contributing to CMD progression [2]. Although ER stress-mediated apoptosis is well characterized in vascular biology [39], CMD-specific human microvascular validation remains indirect.

## 6. Crosstalk Between Oxidative Stress, Regulated Cell Death, and Inflammation

### 6.1. Oxidative Stress as a Central Integrator

Oxidative stress links mitochondrial dysfunction, inflammasome activation, lipid peroxidation, and endothelial injury, thereby acting as a common upstream driver of multiple CMD-related pathways [12,14,15,24].

### 6.2. Interconnection Between Regulated Cell Death Pathways

Regulated cell death pathways do not operate independently but form an interconnected network linked by shared mediators including ROS, inflammatory cytokines, lipid peroxidation products, and DAMPs [12,14,15,62].

### 6.3. ER Stress as a Node of Integration

ER stress links metabolic overload and oxidative stress to multiple regulated cell death pathways [32]. Chronic activation of the unfolded protein response promotes inflammatory signaling and reduces cellular resilience, thereby lowering the threshold for endothelial injury and microvascular deterioration [39].

### 6.4. Self-Reinforcing Loops Driving CMD Progression

At a systems level, metabolic stress, oxidative injury, inflammation, and regulated cell death interact in self-perpetuating networks that sustain endothelial dysfunction and microvascular remodeling [12]. These interconnected pathways promote capillary rarefaction, impair coronary flow reserve, and contribute to CMD progression independent of epicardial atherosclerosis [6]. Consequently, therapeutic strategies targeting shared upstream mechanisms—including redox imbalance, inflammatory signaling, mitochondrial dysfunction, and metabolic dysregulation—may offer broader protection than approaches directed at individual downstream pathways [2]. Collectively, these intersecting immunometabolic, redox, mitochondrial, and regulated cell death pathways form a self-reinforcing network converging on endothelial dysfunction, microvascular remodeling, and impaired CFR. The principal mechanistic axes are summarized in Table 1.

## 7. Comorbidities as Molecular Amplifiers of CMD

### 7.1. Diabetes Mellitus

DM is a major systemic amplifier of CMD through intersecting metabolic, redox, inflammatory, and microstructural mechanisms [17]. Hyperglycemia increases mitochondrial electron transport flux and ROS generation, while activation of the polyol pathway, protein kinase C (PKC) signaling, and the hexosamine pathway further amplifies oxidative stress and endothelial inflammation [17]. Advanced glycation end products (AGEs) activate the receptor for AGEs (RAGE), triggering NF-κB-dependent transcription and sustaining pro-inflammatory cytokine signaling that directly impairs endothelial function [17].

At the endothelial level, DM reduces NO bioavailability via eNOS uncoupling, BH4 depletion, and increased asymmetric dimethylarginine (ADMA), shifting the balance toward vasoconstriction, leukocyte adhesion, and impaired vasodilatory reserve [12,20]. These processes converge with impaired autophagy and mitochondrial quality control to sustain ROS production and inflammasome priming, linking metabolic overload to inflammatory amplification and microvascular barrier vulnerability [14,24].

Clinically, quantitative perfusion imaging demonstrates a high prevalence of reduced CFR/MFR, even in patients without overt obstructive coronary artery disease. In asymptomatic type 2 diabetes, PET-derived MFR is frequently reduced, particularly in the presence of albuminuria, suggesting early coronary microvascular involvement preceding clinical events [66]. Broader evidence supports CMD as a prevalent feature of diabetes, with mechanistic links to endothelial dysfunction and adverse outcomes [67]. Collectively, these data position DM as a prototypical immunometabolic comorbidity that accelerates CMD progression through persistent oxidative stress, inflammatory activation, and microvascular remodeling [12,17].

### 7.2. Obesity and Adipose Tissue Inflammation

Obesity amplifies CMD through chronic low-grade inflammation, adipokine imbalance, and dysfunctional perivascular and epicardial adipose tissue (PVAT/EAT) signaling [18]. In obesity, PVAT shifts from an anti-contractile phenotype toward a pro-inflammatory secretome enriched in TNF-α, IL-6, leptin, resistin, and free fatty acids, promoting endothelial activation, oxidative stress, and impaired vasodilatory capacity [18,65]. Reduced adiponectin removes a key anti-inflammatory and endothelium-protective signal, weakening metabolic resilience and facilitating endothelial dysfunction [18].

Mechanistically, adipose-derived cytokines increase NOX-driven ROS generation, promote NF-κB and STAT signaling, and intensify ER stress and mitochondrial dysfunction, creating conditions permissive for endothelial attrition and microvascular rarefaction [12,39]. Obesity-related systemic lipotoxicity and oxidative lipid stress may also lower the threshold for ferroptosis-like membrane injury in the setting of iron availability and impaired antioxidant buffering [15,16].

Human data link excess adiposity with broad microvascular dysfunction and cardiovascular risk signatures consistent with a systemic microvascular phenotype that plausibly extends to the coronary circulation [68,69]. Focused cardiovascular evidence indicates that obesity is associated with coronary microvascular abnormalities through adipose-mediated inflammatory and vasomotor mechanisms [5,65]. Together, these findings position obesity as a comorbidity that amplifies CMD through endocrine-inflammatory signaling and local perivascular microenvironment remodeling [18,65].

### 7.3. Chronic Kidney Disease

CKD is strongly associated with coronary microvascular dysfunction, even in the absence of flow-limiting epicardial stenosis, supporting a microvascular contribution to the excess cardiovascular risk observed across CKD stages [70,71]. Uremic toxins (e.g., indoxyl sulfate, p-cresyl sulfate) promote endothelial dysfunction through NOX activation, mitochondrial stress, reduced NO bioavailability, and sustained inflammatory signaling [72]. CKD also alters mineral metabolism and increases vascular stiffness, potentially worsening microvascular hemodynamic stress and impairing vasodilatory reserve [72].

Quantitative perfusion studies demonstrate reduced CFR in early CKD, indicating that microvascular dysfunction may occur early in the renal disease trajectory [70]. Importantly, CFR is prognostically meaningful in CKD: PET-derived CFR predicts cardiovascular mortality regardless of CKD stage [71]. A systematic review and meta-analysis confirms significantly lower CFR in CKD versus non-CKD populations and reports a linear association between eGFR and CFR [73].

Beyond vasomotor impairment, CKD is associated with impaired vascular repair capacity and heightened oxidative and inflammatory burden, mechanisms that accelerate microvascular rarefaction and amplify the regulated cell death network described earlier [12,24,49]. CKD should therefore be considered a high-risk CMD amplifier warranting integrated phenotyping and targeted risk reduction strategies [71,73].

### 7.4. Autoimmune Diseases

Autoimmune disorders such as systemic lupus erythematosus (SLE), rheumatoid arthritis, and vasculitis increase cardiovascular risk independently of traditional factors [74]. Chronic immune activation increases inflammatory burden and contributes to endothelial dysfunction, thereby amplifying CMD progression even when epicardial atherosclerosis is not severe [9]. Complement activation recruits leukocytes and amplifies inflammatory injury, bridging innate immunity and vascular damage [75].

In SLE, PET studies demonstrate a high prevalence of CMD (reduced MFR) in symptomatic patients without obstructive CAD, and importantly, the severity of microvascular impairment appears disproportionate to nonobstructive atherosclerotic burden and common risk factors [76]. These findings support CMD as a mechanistic substrate for INOCA-like presentations in autoimmune populations and highlight inflammation as an independent CMD driver [76]. Autoimmune-driven endothelial injury plausibly intersects with immunothrombosis, where complement activation and inflammatory cell–platelet interactions reinforce microvascular obstruction, oxidative stress, and downstream cell-death susceptibility [63,75].

### 7.5. Integrative Perspective

Across diabetes, obesity, CKD, and autoimmune diseases, a shared molecular signature emerges: oxidative stress amplification, impaired NO bioavailability, mitochondrial dysfunction, ER stress activation, and persistent inflammatory priming [12,17,39]. Systemic comorbidities act as accelerators of pre-existing microvascular vulnerability, highlighting the necessity of integrated therapeutic approaches in CMD [2]. Targeting both systemic metabolic inflammation and local microvascular stress may offer synergistic benefits for preserving coronary microvascular integrity.

## 8. Immunothrombosis and Intercellular Signaling in Coronary Microvascular Dysfunction

### 8.1. Immunothrombosis at the Coronary Microvascular Interface

Immunothrombosis represents a physiological mechanism linking innate immunity with coagulation, but it becomes maladaptive in chronic cardiovascular disease states [63]. Neutrophil extracellular traps (NETs) can serve as scaffolds for platelet adhesion and fibrin deposition and contribute to endothelial injury [64]. Complement activation recruits leukocytes and amplifies inflammatory injury, bridging innate immunity and vascular damage [75].

Thrombin signaling through protease-activated receptors (PARs) promotes vascular inflammation and can influence endothelial responses relevant to microvascular dysfunction [77]. Experimental and translational studies suggest that interactions among NETs, complement pathways, platelets, and coagulation factors may contribute to microvascular obstruction and inflammatory vascular remodeling [63,64,75].

Clinically, these processes may contribute to microthrombotic obstruction and impaired coronary flow reserve, especially in inflammatory and prothrombotic CMD phenotypes [6]. However, direct demonstration and quantification of immunothrombotic microvascular obstruction in human CMD remain limited, and much of the currently available evidence originates from experimental vascular models, inflammatory disease studies, and indirect clinical observations rather than dedicated investigations of human coronary microvessels [6,63,64,65,66,67,68,69,70,71,72,73,74,75].

Consequently, immunothrombosis should currently be regarded as a biologically plausible and translationally supported contributor to CMD, although its precise role in human coronary microvascular pathology requires further validation.

### 8.2. Extracellular Vesicles and Intercellular Communication

Extracellular vesicles (EVs), including exosomes, microvesicles, and apoptotic bodies, propagate oxidative stress, inflammation, and prothrombotic signaling across endothelial cells, vascular smooth muscle cells, platelets, and immune cells [78]. The MISEV2023 framework standardizes EV definitions and methodological quality, supporting translational development of EV biomarkers and improving reproducibility across studies [78].

Experimental and translational studies suggest that EVs can influence vascular inflammation, endothelial activation, and thromboinflammatory responses through the transfer of proteins, lipids, and regulatory nucleic acids [78,79]. Accordingly, circulating EV profiles have been proposed as potential biomarkers of vascular dysfunction and cardiovascular risk, supporting their possible role in patient phenotyping and risk stratification.

However, the majority of evidence linking EVs to coronary microvascular dysfunction remains indirect, and prospective clinical studies specifically validating EVs as diagnostic, prognostic, or therapeutic tools in CMD are currently lacking. Consequently, EVs should be regarded as promising but still investigational mediators and biomarkers of microvascular injury. Likewise, therapeutic modulation of EV release, cargo composition, or cellular uptake remains an emerging research direction that requires further clinical validation before translation into routine CMD management [78,79].

### 8.3. Prognostic Implications

CMD predicts adverse cardiovascular outcomes independently of epicardial coronary disease. Reduced CFR/MFR has consistently been associated with increased mortality and adverse cardiovascular event risk, underscoring its value as an integrative functional and prognostic marker of coronary microvascular dysfunction [6,7]. Evidence also indicates that global CFR modifies risk and influences outcomes across a spectrum of obstructive and non-obstructive CAD phenotypes [7].

### 8.4. Therapeutic Targeting

Therapeutic strategies in CMD aim to modulate inflammation, metabolic imbalance, oxidative stress, mitochondrial dysfunction, and thromboinflammatory pathways [2]. However, it should be emphasized that most of the therapeutic approaches discussed below have not been specifically validated in randomized clinical trials conducted in patients with phenotypically characterized CMD. Much of the available evidence derives from broader cardiovascular populations, translational studies, or experimental models and should therefore be interpreted as indirect support for potential CMD-targeted interventions.

Anti-inflammatory targeting has shown clinical benefit in selected atherosclerotic populations. In the CANTOS trial, IL-1β inhibition reduced recurrent cardiovascular events, providing proof-of-concept that inflammatory pathway modulation can improve cardiovascular outcomes [40,80]. However, these benefits were accompanied by an increased incidence of serious infections and infection-related mortality, highlighting the limitations of broad cytokine inhibition and underscoring the need for more selective immunomodulatory strategies [49,80]. Therefore, while inflammation represents an attractive therapeutic target in CMD, direct evidence supporting anti-inflammatory therapy in CMD-specific populations remains limited. These findings support the hypothesis that effective therapeutic strategies may need to balance suppression of maladaptive inflammation with preservation of protective immune surveillance and host defense mechanisms; however, this concept remains to be validated in dedicated CMD studies [59,81].

Metabolic modulation has also gained importance. SGLT2 inhibitors have demonstrated cardiovascular and renal benefits in outcome trials and meta-analyses, supporting their role in cardiometabolic risk reduction and potentially CMD-relevant systemic effects [82]. The EMPA-REG OUTCOME trial provided robust evidence of cardiovascular benefit with empagliflozin in high-risk patients with type 2 diabetes, further supporting metabolic–vascular coupling as a therapeutic axis [83]. Nevertheless, the effects of SGLT2 inhibitors on coronary microvascular function have not yet been conclusively established in dedicated CMD trials.

Precision therapy requires integrating functional imaging, inflammatory biomarkers, and comorbidity profiling to identify patients most likely to benefit from targeted interventions. Given the biological heterogeneity of CMD, therapeutic strategies should be aligned with dominant molecular and microvascular endotypes, while future randomized studies are needed to determine whether mechanism-based interventions may improve coronary microvascular function and clinical outcomes.

### 8.5. Clinical Endotypes and Phenotype-Based Stratification in CMD

The heterogeneity of these thromboinflammatory and immunometabolic mechanisms is mirrored by distinct clinical phenotypes, which arise from different underlying microvascular endotypes. CMD represents a heterogeneous clinical entity within the spectrum of ANOCA/INOCA and CCSs, encompassing both functional and structural endotypes [1,2,3]. Contemporary ESC guidance recognizes that ischemia without obstructive coronary artery disease frequently reflects microvascular and vasomotor abnormalities rather than epicardial stenosis alone [1].

CMD and INOCA are more prevalent in women, reflecting sex-dependent differences in endothelial signaling, hormonal regulation, mitochondrial function, and inflammatory activation [1,3,6]. Post-menopausal hormonal changes may further promote oxidative stress, endothelial senescence, and vasomotor dysfunction, supporting the need for sex-informed CMD phenotyping and management [20,21,24].

The functional microvascular endotype is characterized by impaired vasodilatory reserve or abnormal vasoconstrictive responses in the absence of overt structural rarefaction. This endotype may manifest as reduced CFR with preserved resting flow or as abnormal acetylcholine responses during invasive coronary function testing [3,6]. Mechanistically, this endotype aligns predominantly with nitric oxide signaling failure, redox imbalance, mechanotransduction disturbances, and immunometabolic activation without fixed capillary loss.

In contrast, the structural microvascular endotype is associated with capillary rarefaction, perivascular fibrosis, endothelial attrition, and impaired maximal hyperemic flow [2,6]. This phenotype likely reflects cumulative mitochondrial dysfunction and endothelial attrition driven by ER stress-mediated apoptosis, pyroptosis-like injury, and ferroptosis-associated lipid peroxidation.

A vasospastic or inflammatory overlap phenotype may further integrate endothelial instability, thromboinflammatory signaling, and heightened redox sensitivity, particularly in INOCA populations [3].

Contemporary CMD phenotyping increasingly relies on coronary function testing to distinguish functional from structural microvascular impairment and to guide phenotype-aligned therapeutic strategies. Invasive assessment incorporating CFR, index of microcirculatory resistance (IMR), resistance reserve ratio (RRR), and acetylcholine testing enables endotype differentiation, whereas PET-derived CFR/MFR and stress CMR provide integrative and prognostically robust measures of global microvascular function [6,7]. Importantly, reduced CFR/MFR represents the functional convergence point of diverse molecular perturbations affecting coronary microvascular physiology.

**Figure 2:**
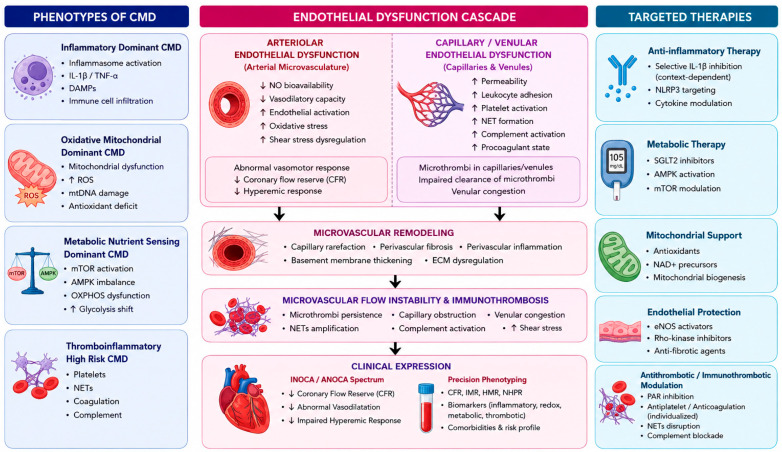
Systems-Level Integration of Molecular Axes and Clinical Endotypes for Precision Therapeutic Stratification in Coronary Microvascular Dysfunction. AMPK—AMP-activated protein kinase; CFR—coronary flow reserve; CMD—coronary microvascular dysfunction; DAMPs—damage-associated molecular patterns; eNOS—endothelial nitric oxide synthase; IL-1β—interleukin-1β; mTOR—mechanistic target of rapamycin; mtDNA—mitochondrial DNA; NAD^+^—nicotinamide adenine dinucleotide; NETs—neutrophil extracellular traps; NO—nitric oxide; NOX—NADPH oxidase; OXPHOS—oxidative phosphorylation; PAR—protease-activated receptor; ROS—reactive oxygen species; SGLT2—sodium–glucose cotransporter 2; TNF-α—tumor necrosis factor-α.

Figure 2 integrates these molecular axes with clinical expression, proposing a phenotype-based framework in which dominant immunometabolic, redox, mitochondrial, or thromboinflammatory endotypes correspond to distinct functional or structural CMD patterns and may guide precision-targeted therapeutic strategies. This integrative approach reframes CMD not merely as a diagnostic category but as a biologically stratifiable vascular disorder with distinct therapeutic implications.

## 9. Future Directions: Toward Systems-Level and Precision Microvascular Medicine

### 9.1. Single-Cell and Spatial Omics in CMD

Advancing the understanding of CMD requires a shift from reductionist approaches to systems-level frameworks. Single-cell technologies enable detailed mapping of vascular and immune cell states, supporting mechanistic dissection of cellular heterogeneity relevant to microvascular disease [81]. Such approaches can identify endothelial and immune subpopulations with distinct metabolic and inflammatory signatures, potentially enabling precision phenotyping in CMD.

### 9.2. Multi-Omics and Computational Modeling

Combining multi-omics datasets with imaging and circulating biomarkers supports the definition of patient-specific CMD phenotypes. Immunometabolism provides a mechanistic basis for integrating metabolic and inflammatory signals across cell types [10]. Computational modeling can help identify dominant pathway signatures—such as immunometabolic activation, oxidative stress predominance, or thromboinflammatory activity—supporting predictive stratification and personalized therapy selection [2].

### 9.3. Longitudinal Phenotyping and Disease Trajectory

Longitudinal studies integrating molecular profiling, functional imaging, and biomarker monitoring are essential to track progression from early endothelial dysfunction to structural microvascular deterioration. Outcome data linking coronary vascular dysfunction to mortality provide a clinical anchor for longitudinal CMD phenotyping [6]. Serial CFR/MFR assessment combined with biomarker profiling may provide dynamic insight into disease activity and therapeutic response in CMD [7].

### 9.4. Translational Implications

The integration of multi-scale data provides opportunities for precision cardiovascular medicine. Therapeutic interventions could be tailored to patients with dominant inflammatory phenotypes (supported by anti-inflammatory outcome evidence) [57] or those with cardiometabolic vulnerability (supported by SGLT2 inhibitor outcomes) [83]. Functional imaging combined with molecular biomarkers may enable monitoring of therapeutic efficacy at tissue and systemic levels, supporting individualized CMD management within CCSs [2].

### 9.5. Integrative and Systems-Level Framework

CMD should be conceptualized as a network disease characterized by interconnected pathways of oxidative stress, metabolic dysregulation, inflammation, regulated cell death, and immunothrombosis. A systems-level approach emphasizes multidimensional assessment, combining molecular phenotyping, functional imaging, and computational modeling to guide precision interventions [2]. Ultimately, this framework may redefine CCS management, shifting from a focus on epicardial stenosis to a comprehensive strategy addressing systemic and microvascular dysfunction simultaneously [1].

## 10. Limitations of Current Evidence

Although substantial progress has been made in understanding the molecular basis of CMD, important limitations remain. Several mechanisms discussed in this review, including ferroptosis, extracellular vesicle signaling, and immunothrombosis, are supported predominantly by experimental or translational evidence, whereas direct validation in human coronary microvascular tissue remains limited. Furthermore, many therapeutic concepts derive from broader cardiovascular populations rather than dedicated CMD trials. Consequently, some pathways should currently be regarded as biologically plausible contributors rather than definitively established drivers of CMD. Future studies integrating molecular phenotyping, advanced imaging, and prospective clinical validation are required to further refine CMD endotypes and therapeutic strategies.

## 11. Conclusions

CMD in CCSs arises from a highly interconnected network of immunometabolic dysregulation, oxidative stress, mitochondrial dysfunction, and regulated cell death [2]. Early endothelial activation is driven by disturbances in nitric oxide signaling and redox imbalance, while metabolic shifts toward glycolytic programs and dysregulated nutrient sensing reinforce pro-inflammatory phenotypes [10].

Mitochondrial injury and impaired quality control amplify reactive oxygen species production, linking metabolic stress to inflammasome activation and inflammatory amplification [24]. Accumulating experimental and translational evidence suggests that regulated cell death—including pyroptosis, ferroptosis-like vulnerability, necroptosis, apoptosis, and ER stress-mediated pathways—contributes to endothelial attrition, capillary rarefaction, and impaired coronary flow reserve [14].

These mechanisms interact in self-reinforcing loops: oxidative stress promotes inflammation, inflammatory mediators exacerbate redox imbalance, and metabolic dysregulation lowers the threshold for cell death [12]. Emerging evidence suggests that immunothrombosis and extracellular vesicle-mediated signaling may propagate injury across local and systemic scales, linking microvascular dysfunction with thromboinflammatory complications [63].

Systemic comorbidities such as diabetes, obesity, chronic kidney disease, and autoimmune disorders amplify these molecular pathways, accelerating CMD progression and worsening prognosis [17]. Reduced CFR/MFR reflects the integrated functional consequence of these molecular and cellular perturbations and remains a robust predictor of adverse cardiovascular outcomes [6].

A systems-level, molecular understanding of CMD reframes CCSs as systemic, immunometabolic vascular disorders rather than merely epicardial diseases [2]. Precision cardiovascular medicine—integrating molecular phenotyping, functional imaging, multi-omics profiling, and targeted modulation of inflammatory, redox, metabolic, and thromboinflammatory pathways—has the potential to preserve microvascular integrity, improve risk stratification, and potentially modify the trajectory of chronic ischemic heart disease [1].

Future research should focus on integrating longitudinal molecular phenotyping with functional assessments, enabling early identification of high-risk CMD phenotypes and guiding personalized therapeutic interventions [83]. Through this approach, CMD management may evolve from generalized anti-atherosclerotic strategies to precision-guided therapies that target the underlying molecular and cellular mechanisms, ultimately improving clinical outcomes in CCS patients [2].

## Figures and Tables

**Table 1:** Integrated Immunometabolic and Regulated Cell Death Axes Driving Coronary Microvascular Dysfunction.

Dominant Pathophysiological Axis	Key Molecular Drivers	Dominant Cellular Effects	Microvascular Structural/Functional Consequence	Functional Impact on Coronary Flow Reserve (CFR)	Candidate Therapeutic Nodes	Related Comorbidities	Current Evidence Base
NO signaling failure	eNOS uncoupling; BH4 depletion; ↑ ADMA; ↓ KLF2/4 [20,21]	↓ NO bioavailability; ↑ superoxide; endothelial activation	Impaired endothelium-dependent vasodilation; increased leukocyte adhesion	Vasodilatory impairment (↓ CFR)	BH4-targeted approaches (experimental); AMPK activation; eNOS recoupling strategies	HTN, diabetes, obesity, aging	Human clinical and mechanistic studies
Oxidative stress amplification	NOX activation; mitochondrial ROS; Nrf2 dysregulation [12,24]	Lipid peroxidation; redox imbalance; inflammatory priming	Endothelial dysfunction; reduced adaptive redox buffering	Hyperemic limitation (↓ CFR)	Nrf2 activation; redox-targeted therapies	Diabetes, CKD, obesity	Human clinical and mechanistic studies
Immunometabolic reprogramming	HIF-1α stabilization; succinate; PKM2; mTORC1 hyperactivation [10,34]	Glycolytic shift; IL-1β induction; metabolic inflexibility	Sustained endothelial inflammation; impaired metabolic adaptability	Inflammation-driven vasomotor impairment (↓ CFR)	AMPK activation; mTOR modulation; IL-1β inhibition	Diabetes, obesity, metabolic syndrome	Translational and mechanistic studies
Mitochondrial dysfunction	DRP1-mediated fission; NAD^+^ depletion; impaired mitophagy [36,42]	Bioenergetic failure; ROS overproduction	Reduced microvascular resilience; impaired flow-mediated dilation	Energy-dependent vasomotor limitation (↓ CFR)	NAD^+^ augmentation; mitochondrial quality control strategies	Aging, HF, diabetes	Predominantly translational studies
ER stress/UPR activation	PERK; IRE1α; ATF6 [39]	Pro-apoptotic signaling; endothelial apoptosis susceptibility; inflammatory amplification	Capillary rarefaction; endothelial barrier instability	Structural flow limitation (↓ CFR)	Autophagy enhancement; ER stress modulation	Diabetes, obesity	Predominantly translational studies
Pyroptosis	Inflammasome activation; caspase-1; gasdermin D [14,55]	Membrane pore formation; IL-1β/IL-18 release	Endothelial barrier disruption; microvascular injury	Inflammatory capillary dysfunction (↓ CFR)	Inflammasome inhibition; IL-1β targeting (clinical proof-of-concept)	Diabetes, autoimmune disease	Experimental and translational studies
Ferroptosis-like vulnerability	Iron accumulation; lipid peroxidation; GPX4 axis impairment [15,16]	Oxidative membrane damage; regulated necrotic death	Endothelial attrition; microvascular loss	Rarefaction-associated perfusion decline (↓ CFR)	Iron modulation; lipid peroxidation control	CKD, diabetes	Predominantly experimental; indirect human observations
Necroptosis	RIPK1/3 signaling [62]	Lytic cell death; DAMP release	Amplified local inflammation; microvascular structural injury	Inflammation-mediated flow instability (↓ CFR)	RIPK pathway modulation (experimental)	Autoimmune disease	Predominantly experimental studies
Immunothrombosis	NETs; complement activation; thrombin; PAR signaling [63,64]	Microthrombus formation; platelet–leukocyte interaction	Capillary flow obstruction at the microvascular level	Microthrombotic perfusion restriction (↓ CFR)	Antithrombo-inflammatory strategies (e.g., NET inhibition—preclinical)	Autoimmune disease, CKD	Translational studies and indirect clinical evidence
Adipose–vascular cross-talk	EAT/PVAT cytokines (TNF-α, IL-6); lipotoxic mediators [18,65]	Chronic low-grade inflammation; endothelial activation	Microvascular remodeling; impaired vasomotor tone	Metabolic-inflammatory flow dysregulation (↓ CFR)	Metabolic therapies (e.g., SGLT2 modulation)	Obesity, metabolic syndrome	Human observational and translational studies

ADMA—asymmetric dimethylarginine; AMPK—AMP-activated protein kinase; ATF6—activating transcription factor 6; BH4—tetrahydrobiopterin; CFR—coronary flow reserve; DAMPs—damage-associated molecular patterns; DRP1—dynamin-related protein 1; EAT—epicardial adipose tissue; eNOS—endothelial nitric oxide synthase; ER—endoplasmic reticulum; GPX4—glutathione peroxidase 4; HIF-1α—hypoxia-inducible factor-1α; IRE1α—inositol-requiring enzyme-1α; KLF2/4—Krüppel-like factor 2/4; mTORC1—mechanistic target of rapamycin complex 1; NAD^+^—nicotinamide adenine dinucleotide; NETs—neutrophil extracellular traps; NOX—NADPH oxidase; Nrf2—nuclear factor erythroid 2-related factor 2; PAR—protease-activated receptor; PERK—protein kinase R-like endoplasmic reticulum kinase; PKM2—pyruvate kinase M2; PVAT—perivascular adipose tissue; RIPK—receptor-interacting protein kinase; ROS—reactive oxygen species; SGLT2—sodium–glucose cotransporter 2; TNF-α—tumor necrosis factor-α; UPR—unfolded protein response.

## Data Availability

No new data were created or analyzed in this study. Data sharing is not applicable to this article.

## Abbreviations

ADMA	asymmetric dimethylarginine
AGEs	advanced glycation end products
AMPK	AMP-activated protein kinase
ATF6	activating transcription factor 6
BH4	tetrahydrobiopterin
CAD	coronary artery disease
CANTOS	Canakinumab Anti-inflammatory Thrombosis Outcomes Study
CCSs	chronic coronary syndromes
CDKN2A	cyclin-dependent kinase inhibitor 2A (p16^INK4a)
CFR	coronary flow reserve
cGAS	cyclic GMP–AMP synthase
CKD	chronic kidney disease
CMD	coronary microvascular dysfunction
DAMPs	damage-associated molecular patterns
DM	diabetes mellitus
DRP1	dynamin-related protein 1
EAT	epicardial adipose tissue
eGFR	estimated glomerular filtration rate
eNOS	endothelial nitric oxide synthase
ER	endoplasmic reticulum
EVs	extracellular vesicles
FAK	focal adhesion kinase
GPX4	glutathione peroxidase 4
HIF-1α	hypoxia-inducible factor-1α
IL	interleukin
IMR	index of microcirculatory resistance
INOCA	ischemia with non-obstructive coronary arteries
IRE1α	inositol-requiring enzyme-1α
KLF2/4	Krüppel-like factor 2/4
MAPK	mitogen-activated protein kinase
MFR	myocardial flow reserve (PET-derived term corresponding to CFR)
MISEV2023	Minimal Information for Studies of Extracellular Vesicles 2023
mTOR	mechanistic target of rapamycin
mTORC1	mechanistic target of rapamycin complex 1
mtDNA	mitochondrial DNA
NAD^+^	nicotinamide adenine dinucleotide
NETs	neutrophil extracellular traps
NF-κB	nuclear factor kappa B
NO	nitric oxide
NOX	NADPH oxidase
Nrf2	nuclear factor erythroid 2-related factor 2
OXPHOS	oxidative phosphorylation
PARs	protease-activated receptors
PERK	protein kinase R-like endoplasmic reticulum kinase
PET	positron emission tomography
PI3K–Akt	phosphoinositide 3-kinase–Akt signaling pathway
PKC	protein kinase C
PVAT	perivascular adipose tissue
RAGE	receptor for advanced glycation end products
RIPK	receptor-interacting protein kinase
ROS	reactive oxygen species
RRR	resistance reserve ratio
SASP	senescence-associated secretory phenotype
SGLT2	sodium–glucose cotransporter 2
SLE	systemic lupus erythematosus
STAT3	signal transducer and activator of transcription 3
STING	stimulator of interferon genes
TCA	tricarboxylic acid cycle
TNF-α	tumor necrosis factor-α
UPR	unfolded protein response
